# Prognostic value of nuclear morphometry in patients with TNM stage T1 ovarian clear cell adenocarcinoma

**DOI:** 10.1038/sj.bjc.6690276

**Published:** 1999-04

**Authors:** C Q Liu, H Sasaki, M T Fahey, A Sakamoto, S Sato, T Tanaka

**Affiliations:** Department of Obstetrics & Gynecology, The Jikei University School of Medicine, 3-25-8 Nishi-shinbashi, Minato-ku, Tokyo, 105, Japan; Epidemiology & Biostatistics Division, National Cancer Center, Research Institute East, 6-5-1 Kashiwanoha, Chiba, 277, Japan; Department of Pathology, University of Tokyo, Faculty of Medicine, Hongo, Tokyo, 113, Japan; and; Department of Obstetrics & Gynecology, Tohoku University School of Medicine, 2-1, Seiryocho, Sendai, 980-99, Japan

**Keywords:** ovarian clear cell adenocarcinoma, nuclear morphometry, TMN stage T1, prognosis, lymphatic involvement

## Abstract

In 40 patients with TNM stage T1 ovarian clear cell adenocarcinoma, we used nuclear morphometry to study the relations among morphometric variables, clinical prognostic factors and outcome. The presence of one or more giant nuclear cells was positively associated with death (OR = 10.6, *P* = 0.02) and tended to be associated with disease recurrence (OR = 5.1, *P* = 0.07). Nuclear irregularity (expressed in terms of the nuclear roundness factor) was positively associated with both death (OR = 8.6, *P* = 0.02) and disease recurrence (OR = 8.2, *P* = 0.02). A combination of giant nuclear cell presence or nuclear irregularity proved to be a useful prognostic indicator, with a sensitivity and specificity of 83% and 71% in the prediction of death, and 75% and 71% in the prediction of disease recurrence. Patients' age and substage were of no prognostic value. We conclude that the nuclear morphometric characteristics, especially the presence of giant nuclear cells and nuclear irregularity, may be useful in predicting outcome in patients with early stage ovarian clear cell adenocarcinoma. © 1999 Cancer Research Campaign


*British Journal of Cancer* (1999) **79**(11/12), 1736–1741
© 1999 Cancer Research Campaign
Article no. bjoc.1998.0276

### Prognostic value of nuclear morphometry in patients with TNM stage T1 ovarian clear cell adenocarcinoma

**CQ Liu[1], H Sasaki[1], MT Fahey[2], A Sakamoto[3], S Sato[4] and T Tanaka[1]**

[1]Department of Obstetrics & Gynecology, The Jikei University School of Medicine, 3-25-8 Nishi-shinbashi, Minato-ku, Tokyo, 105, Japan; [2]Epidemiology & Biostatistics Division, National Cancer Center, Research Institute East, 6-5-1 Kashiwanoha, Kashiwa-shi, Chiba, 277, Japan; [3]Department of Pathology, University of Tokyo, Faculty of Medicine, Hongo, Bunkyo-ku, Tokyo, 113, Japan; and [4]Department of Obstetrics & Gynecology, Tohoku University School of Medicine, 2-1, Seiryocho, Aoba-ku, Sendai, 980-99, Japan

**Summary** In 40 patients with TNM stage T1 ovarian clear cell adenocarcinoma, we used nuclear morphometry to study the relations among morphometric variables, clinical prognostic factors and outcome. The presence of one or more giant nuclear cells was positively associated with death (OR = 10.6, $P = 0.02$) and tended to be associated with disease recurrence (OR = 5.1, $P = 0.07$). Nuclear irregularity (expressed in terms of the nuclear roundness factor) was positively associated with both death (OR = 8.6, $P = 0.02$) and disease recurrence (OR = 8.2, $P = 0.02$). A combination of giant nuclear cell presence or nuclear irregularity proved to be a useful prognostic indicator, with a sensitivity and specificity of 83% and 71% in the prediction of death, and 75% and 71% in the prediction of disease recurrence. Patients' age and substage were of no prognostic value. We conclude that the nuclear morphometric characteristics, especially the presence of giant nuclear cells and nuclear irregularity, may be useful in predicting outcome in patients with early stage ovarian clear cell adenocarcinoma.

**Keywords:** ovarian clear cell adenocarcinoma; nuclear morphometry; TMN stage T1; prognosis; lymphatic involvement

Ovarian clear cell adenocarcinoma (OCCA) has been recognized as a distinct histological entity in the World Health Organization (WHO) classification of ovarian tumours since 1973 (Serov et al, 1973). OCCA constitutes 8% to 10% of all ovarian epithelial malignancies (Kennedy et al, 1989) and has a worse prognosis than other types of ovarian cancer (Jenison et al, 1989; Goff et al, 1996). Although the outcome of patients with early-stage disease is substantially better than that of their counterparts with advanced-stage disease, a significant proportion of women (20% to 50%) with stage I OCCA have recurrence and die of their malignancy (O'Brien et al, 1993). It would be desirable to have accurate methods to identify patients with early stage OCCA who are at high risk for tumour progression. Histological grade has been shown to be useful as an initial prognostic determinant in some studies of epithelial cancer of the ovary (Haapasalo et al, 1990, 1991; Kennedy et al, 1993), but the histological grading of OCCA is still controversial and complicated by the multiplicity of histological patterns presented in the same tumour. Some previous works have focused on the prognostic value of nuclear grading systems, such as the Fuhrman grading system (Fuhrman et al, 1982) for renal cell carcinoma and Christopherson's criteria (Christopherson et al, 1982) for endometrial clear cell carcinoma. Unfortunately, neither of these grading systems has shown predictive value for survival (Montag et al, 1989).

During the last decade, various investigators have demonstrated the usefulness of nuclear morphometry as a predictor of the clinical behaviour for a wide variety of neoplasms (Sharkey et al, 1983; Colombel et al, 1995; Fukuzawa et al, 1995; Nativ et al, 1995) including ovarian carcinoma (Miller et al, 1991). However, the question remains unresolved as to whether nuclear morphometric parameters may reflect the biological virulence of a particular tumour in the individual. In this study, we measured nuclear variables in 40 patients with OCCA locally restricted to the ovary and studied the relations of these variables to clinical prognostic factors and outcome.

*Received 24 February 1998*
*Revised 18 August 1998*
*Accepted 13 October 1998*

*Correspondence to:* H Sasaki

### MATERIAL AND METHODS

### Patients

Between January 1988 and March 1996, a total of 168 consecutive patients with TNM stage T1 (defined as intra-abdominal disease confined to one or both ovaries) invasive epithelial ovarian cancer were treated at the Department of Obstetrics & Gynecology of the Jikei University School of Medicine Hospital, Tokyo, Japan. Among them, 40 (23.8%) patients were found to have pure ovarian clear cell adenocarcinoma. Patients were staged by the TNM staging system and the local diseases were substaged according to the 1986 revised staging system of the International Federation of Gynecology & Obstetrics. All patients underwent a surgical staging procedure consisting of a total abdominal hysterectomy and bilateral salpingo-oophorectomy in addition to an infracolic omentectomy, peritoneal cytological washings, and biopsies of all places in the abdominal cavity at risk for tumour metastasis. Twenty-one patients underwent pelvic and paraoartic lymphadenectomy. The selection of patients for lymphadenectomy was influenced by surgical findings, the medical condition, anaesthetic considerations and the skill of the surgeon. All patients received adjuvant therapy consisting of six courses of a combination of cisplatin, adriamycin and cyclophosphamide (CAP). Follow-up data were obtained by reviewing hospital records. Survival was considered the primary end-point and survival time was measured from the date of diagnosis to the date of death or last contact. The deaths recorded in the study all were related directly to carcinoma.

### Morphometric analysis

The samples were fixed in 10% buffered neutral formalin and embedded in paraffin. Sections of 5-μm thickness were cut and stained with haematoxylin and eosin. One experienced gynaecological pathologist (AS) reviewed all tumour slides and selected two identical slides for each case. The slides were coded by consecutive numbers, and all histological and morphometric analyses were performed without knowledge of patient identity and outcome.

Five photographs were taken with high-resolution black-and-white film at × 1150 magnification with an Olympus Microscope. The cell-field images were captured by the operator, who moved the microscopic fields randomly across the specimen. Areas with inflammation and necrosis (if present) were carefully avoided. Each photograph included 30 to 60 cells. All clear-cell adenocarcinoma nuclei within the chosen field that could be focused sharply were traced. The images were processed with an IBM-compatible personal computer, an imaging screen, and image measurement software (Image Command 5098, version 2.43). From each tumour, the characteristics of about 160 to 350 nuclei were measured. Fifty of these nuclei were randomly selected with the use of a computer-generated list of random numbers for further analysis. For each nucleus, four variables were measured directly: nuclear area (NA); nuclear longer diameter (NLD); nuclear perimeter (NP); and the nuclear roundness factor (NRF). The NRF was defined as the degree to which the nucleus in cross-section approximated a perfect circle. If the nucleus was a perfect circle in cross-section then it was assigned a roundness factor of 1.00. As the nuclear shape deviates from that of a perfect circle, values of NRF depart from 1.00. NRF was computed from the following formula: nuclear roundness factor = M.NP/I.NP, where M.NP is the measured perimeter, and I.NP is the perimeter of an imaginary perfect circle with an area equivalent to the measured nuclear area.

### Statistical analysis

The distribution of each morphometric variable was examined as a histogram for each patient according to survival and disease recurrence status. Because of a positive skew in the data for patients who died, the median was used to describe each patient's distribution. Nuclear irregularity was defined as present if the patient median NRF was greater than or equal to 1.04, which was the 75th percentile of all patient median NRF values. A giant nuclear cell (GNC) was defined as any cell with an NLD greater than or equal to twice the median NLD for a given patient. Between-patient differences in nuclear morphometry were summarized using the median and interquartile range (IQR) of each patient's median value. Associations between clinical or morphometric factors and death or disease recurrence were measured by sensitivity, specificity and the odds ratio (OR). Conditional maximum likelihood was used to estimate ORs and exact mid-*P* confidence intervals (CIs) (Williams, 1988). The Kaplan–Meier method was used to estimate survival curves and the log-rank statistic to test for a difference between curves. PEPI version 2.05 was used to compute exact mid-*P* values and confidence intervals (Gahlinger and Abramson, 1995) for sensitivity, specificity and the odd ratio. Klotz's linked list method was used to compute exact *P*-values for the Wilcoxon rank sum test (Klotz et al, in press)

### RESULTS

Patient characteristics are shown in Table 1. The median age of the patients was 50 years (IQR 43 to 58 years). Eighteen patients had stage T1a and 22 stage T1c cancer. Two of the 21 patients who underwent lymphadenectomy were found to have pelvic lymph node involvement, and one patient had both pelvic and para-arotic lymph node metastasis. The median follow-up of the six patients who died was 788 days (IQR 644 to 1205 days) and the median follow-up of the remaining patients was 1484 days (IQR 1150 to 2012 days). Eight patients relapsed during the follow-up.

The distribution of NLD for a patient who died 862 days after primary surgery is shown in Figure 1A, and for a patient who was alive at the end of follow-up in Figure 1B. Distributional patterns similar to these figures were seen in many patients and values of NLD were spread over a greater range among patients who died than among those who were alive at the end of follow-up. However, this finding was mainly due to the presence of a small number of very large nuclear cells. Similar distributional patterns were observed in patients with and without disease recurrence and with respect to other variables of cell morphometry (data not shown).

The median and interquartile range of each patient's individual median values were reported according to survival and disease recurrence for each morphometric variable in Table 2. Summary median values were higher among patients who died than among survivors for all of nuclear morphometric variables. Median differences (died vs alive) were statistically significant for NLD (10.5 vs

**Table 1** Characteristics of 40 patients with stage T1 ovarian clear cell carcinoma

| Characteristic | No. of patients (%) |
|---|---|
| Died | 6 (15) |
| Disease recurrence | 8 (20) |
| Age > 50 years old | 20 (50) |
| Substage | |
| T1a | 18 (45) |
| T1c | 22 (55) |
| Lymphadenectomy | 21 (52) |
| Metastasis | 3/21 (14) |
| No metastasis | 18/21 (86) |
| Giant nuclear cell[a] | 9 (22) |
| Nuclear irregularity[b] | 10 (25) |
| Giant nuclear cell or nuclear irregularity | 15 (38) |

[a]Cell with a nuclear longer diameter (NLD) greater than or equal to twice the median NLD. [b]Patient median nuclear roundness factor (NRF) greater than or equal to 1.040.

© Cancer Research Campaign 1999

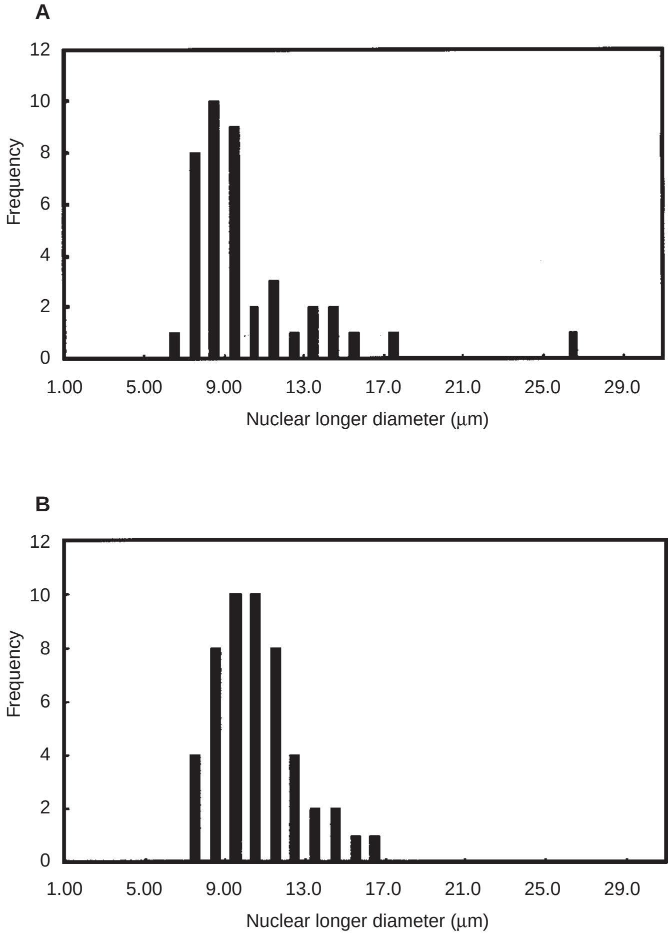

**Figure 1** (**A**) Distribution of nuclear longer diameter from one patient with OCCA who died 862 days after primary surgery. (**B**) Distribution of nuclear longer diameter from one patient with OCCA who showed no evidence of recurrence 2124 days after the primary surgery

**Table 2** Summary of nuclear morphometry measures according to survival and disease recurrence status

| Measure | Case status | | | |
|---|---|---|---|---|
| | **Died (*n* = 6)** | **Alive (*n* = 34)** | **Recurrence (*n* = 8)** | **No recurrence (*n* = 32)** |
| Nuclear area (µm²) | | | | |
| Median | 65.6* | 52.6 | 64.5* | 52.6 |
| Interquartile range | 63.8–69.9 | 47.3–63.9 | 55.3–67.9 | 47.2–66.5 |
| Nuclear longer diameter (µm) | | | | |
| Median | 10.5** | 9.0 | 10.1** | 9.0 |
| Interquartile range | 10.0–10.8 | 8.4–10.2 | 9.5–10.8 | 8.4–10.3 |
| Nuclear perimeter (µm) | | | | |
| Median | 30.2** | 26.7 | 29.6 | 26.7 |
| Interquartile range | 29.6–31.6 | 25.0–30.0 | 27.4–31.1 | 25.0–30.1 |
| Nuclear roundness factor | | | | |
| Median | 1.046*** | 1.028 | 1.044*** | 1.028 |
| Interquartile range | 1.037–1.059 | 1.024–1.038 | 1.034–1.054 | 1.024–1.037 |

*P*-value for comparison with the adjacent column by Wilcoxon rank sum test, *, $P > 0.05$, **, $P \leq 0.05$, ***, $P \leq 0.005$.

9.0 µm, $P = 0.03$), NP (30.2 vs 26.7 µm, $P = 0.05$) and NRF (1.046 vs 1.028, $P < 0.01$), but not for NA (65.6 vs 52.6 µm², $P = 0.07$). A similar pattern was observed among patients with and without disease recurrence, although the differences were smaller, and statistical significance was found only for NLD (10.1 to 9.0 µm, $P = 0.05$) and NRF (1.044 vs 1.024, $P < 0.01$). Of the patients who underwent lymphadenectomy, median values were higher among those with lymph node metastasis than those without

*© Cancer Research Campaign 1999*

**Table 3** Bivariable association between clinical or nuclear morphometry factors and outcome[a]

| | Death | | Disease recurrence | |
|---|---|---|---|---|
| **Factor** | **OR (CI)** | ***P*-value** | **OR (CI)** | ***P*-value** |
| Age > 50 years old | 2.21 (0.34, 19) | 0.422 | 1.86 (0.37, 11) | 0.465 |
| Stage T1c | 0.79 (0.12, 5.2) | 0.804 | 0.78 (0.15, 4.0) | 0.765 |
| Giant nuclear cell | 10.6 (1.5, 102) | 0.017 | 5.1 (0.89, 31) | 0.067 |
| Nuclear irregularity | 8.6 (1.3, 81) | 0.016 | 8.3 (1.5, 55) | 0.016 |
| Giant nuclear cell or nuclear irregularity | 11.2 (1.4, 296) | 0.020 | 7.2 (1.5, 55) | 0.024 |

[a]Exact mid-*P* values and confidence intervals were computed.

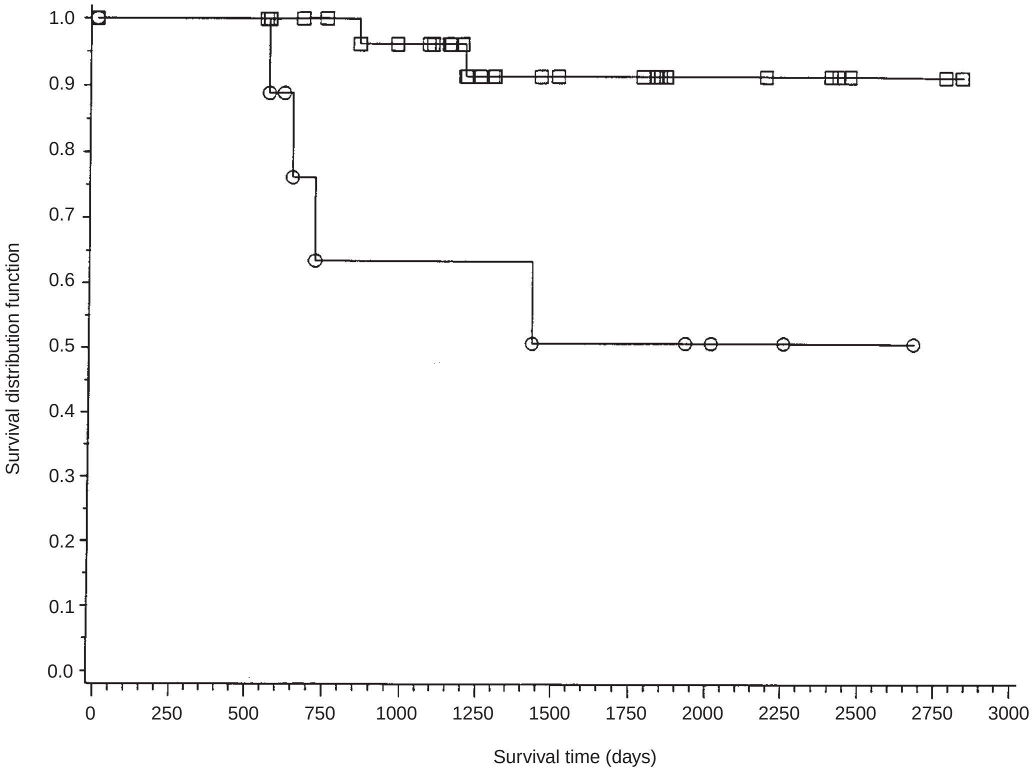

**Figure 2** Survival of patients with or without GNC (giant nuclear cell) groups: -□- no giant nuclear cell; -○- presence of one or more giant nuclear cell metastasis for both NLD (10.3 vs 91 μm, $P$ = 0.03) and NRF (1.044 vs 1.030, $P$ = 0.09).

The association between clinical or morphometric factors and survival or disease recurrence is reported in Table 3. The presence of one or more GNCs was positively associated with death (OR = 10.6, $P$ = 0.02) and tended to be associated with disease recurrence (OR = 5.1, $P$ = 0.07). Nuclear irregularity was positively associated with both death (OR = 8.6, $P$ = 0.02) and disease recurrence (OR = 8.3, $P$ = 0.02). The presence of GNC or nuclear irregularity in combination was associated with both death (OR = 11.2, $P$ = 0.02) and disease recurrence (OR = 7.2, $P$ = 0.02). The sensitivity and specificity of the presence of GNC or nuclear irregularity were 83% (95% CI: 41, 99) and 71% (95% CI: 54, 84) for the prediction of death, and 75% (95% CI: 56, 89) and 71% (95% CI: 54, 84) for the prediction of recurrence, respectively. When the sample size was sufficient, ORs for all associations involving either GNC or irregularity were adjusted separately for age and stage. The adjusted results were similar to the crude results and are not reported. Patient age and the substage did not show any association with death or disease recurrence.

Survival of patients without a GNC was better than that of patients with a GNC ($\chi^2$ = 8.0, $P$ < 0.01), as shown in Figure 3. After 19 months the survival rate was 100% in patients without a GNC (first death after 858 days) and 89% for patients with one or more GNCs (first death after 567 days). The 2-year survival rates were 100% and 63%, respectively. Among patients without a

© Cancer Research Campaign 1999 

GNC, the numbers at risk of death at 12, 18 and 24 months were 31, 31 and 28 respectively, as compared with 9, 9 and 5 among patients with one or more GNCs.

### DISCUSSION

The anatomical extent of tumour at diagnosis has been the most important parameter for determining the outcome of patients with epithelial ovarian cancer. Approximately 40% of all patients present with FIGO stage I. The 5-year survival is related to FIGO stage ranging from 82% for FIGO stage Ia to 68% for stage Ic. Several studies have shown that histological grade was the most important predictor of outcome in stage I disease, and for patients with FIGO stage Ia, Ib and well- or moderately differentiated tumours adjuvant therapy after surgery is unnecessary (Dembo et al, 1990; Finn et al, 1992). However, the situation is quite different for OCCA. Comparison of survival for patients with OCCA and papillary serous histology have shown that OCCA is associated with a poorer outcome. In a study by Jenison and colleagues, the estimated 5-year survival for patients with stage I OCCA were 50% compared with 87% for papillary serous carcinoma (Jenison et al, 1989). The same tendency was also found for patients with stage III patients, with a median survival of 12 months compared with 22 months for papillary serous carcinoma (Goff et al, 1996). Moreover, there is no clearly defined, generally accepted and widely used grading system for OCCA. The GOG Pathology Manual published in 1994 did not include a grading system for OCCA (Benda and Zaino, 1994). Our preliminary investigation found that Fuhrman's grading system for renal carcinoma did not stratify effectively the tumours among the four grades, with only 7.5% (3/40) classified as grade 1 or grade 2 (unpublished data). This is in accordance with the data of Kennedy and colleagues (Kennedy et al, 1993). Histoquantitative techniques have generated considerable interest because of their potential to improve subjective grading. Nuclear morphometry attempts to quantify phenotypical heterogeneity in terms of shape alterations, while flow cytometry may identify chromosomal aberrations in terms of changes in the DNA content. Compared with flow cytometry, nuclear morphometry analysis is a simple, objective and inexpensive method for identifying malignant features in tumour cells. Morphometry analysis is performed on routine H&E sections and noncancer nuclei can be avoided. The accuracy of nuclear morphometry analysis may be superior to flow cytometry in the detection of minimal change and rare events (Miller et al, 1991).

An important finding of this study was that the presence of GNC or nuclear irregularity was positively associated with the tumour progress of TNM stage T1 OCCA. For ovarian cancer, the percentage of hyperploid cells (with nuclear DNA content ≥5C) has been found to be significantly higher in grades 2 and 3 than in grade 1 tumours, i.e. in rapidly progressive tumours compared with less aggressive malignancies (Miller et al, 1991). It may identify high-risk patients with ovarian cancer (Wagner et al, 1994). Rodenburg et al (1988) labelled hyperploid cells as 'marker cells' for malignant changes, such as increased genetic instability and impaired growth regulation. Nuclear size has been found to be associated with the DNA content of the cells (Baak et al, 1988) and it is reasonable to assume that the GNCs identified by morphometry in this study are consistent with these observations. While many different nuclear descriptors have been used to summarize the morphology of the nucleus, nuclear size is the most widely used parameter. Giant nuclear cells can be readily identified and appear to be more reproducible.

Nuclear irregularity has long been used by pathologists to be an indicator of malignant potential for various types of tumour. However, such assessment is subjective and poorly reproducible. It was not until 1984 that Diamond et al (1984) first proposed the concept of the nuclear roundness factor as a prognostic indicator for patients with stage B1 and B2 prostatic carcinoma after radical prostatectomy. Partin and colleagues measured 16 different descriptors and they found that nuclear roundness factor was the most useful shape descriptor for the prediction of disease-free interval for patients with stage A2 prostate cancer (Partin et al, 1989). In our present work, nuclear irregularity was positively associated with both death and disease recurrence. This result is in agreement with the studies of Pound et al (1993) on renal cell carcinoma and of Borland et al (1993) on deeply invasive bladder carcinoma.

Pelvic lymph node metastasis has been reported to occur in 8% to 15% and para-aortic lymph node metastasis in 5% to 24% of patients with intra-abdominal stage I ovarian cancer (Petru et al, 1994). FIGO has recommended evaluating retroperitoneal lymph nodes in the staging of ovarian cancer patients. It is still unclear whether patients who receive lymphadenectomy for early-stage ovarian cancer benefit from this procedure (Finn et al, 1992; Petru et al, 1994). The ability to identify patients at high risk for lymphatic involvement would allow us to limit retroperitoneal lymphadenectomy selectively to a subset of patients whose tumours show a high metastatic potential. There were three patients in this study who had pelvic or para-aortic lymph node involvement or both. The result of the nuclear morphometric analysis of these three tumours was consistent with the hypothesis that nuclear size and shape are correlated with the tendency to lymphatic involvement even when the disease is locally confined to the ovary. Although a large study is required to quantify precisely the strength of association between NLD and metastasis potential, our results suggest an hypothesis for further investigation.

The present treatment for ovarian cancer is by no means ideal. One way to obtain a more rational basis for the treatment policy may be better biological characterization of the tumour. The use of several prognostic variables may allow us to tailor treatment to individual patients with OCCA and thereby provide maximal therapeutic benefit with minimal risk. Nuclear morphometry, especially the quantitative, objective measurement of nuclear heterogeneity and nuclear pleomorphism seems to be a step in that direction.

### REFERENCES

Baak JA, Schipper NW, Wisse-Brekelmans ECM, Ceelen T, Bosman FT, van Geuns H and Wils J (1988) The prognostic value of morphometrical features and cellular DNA content in cis-platin-treated late ovarian cancer patients. *Br J Cancer* **57**: 503–508

Benda JA and Zaino R (1994) Histologic classification of tumours of the ovary. In *GOG Pathology Manual*. Gynecologic Oncology Group

Borland RN, Partin AW, Epstein JI and Brendler CB (1993) The use of nuclear morphometry in predicting recurrence of transitional cell carcinoma. *J Urol* **149**: 272–275

Christopherson WM, Alberhasky RC and Connelly PJ (1982) Carcinoma of the endometrium. 1. A clinicopathologic study of clear cell carcinoma and secretory carcinoma. *Cancer* **49**: 1511–1523

Colombel M, Delaunoit Y, Bellot J, Kiss R, Abbou C and Chopin D (1995) Prognostic evaluation of morphonuclear parameters in superficial and invasive bladder cancer. *Br J Urol* **75**: 364–369

 © Cancer Research Campaign 1999

Dembo AJ, Davy M, Stenwig AE and Bush RS (1990) Prognostic factors in patients with stage I epithelial ovarian cancer. *Obstet Gynecol* **75**: 263–273

Diamond DA, Berry SJ, Jewett HJ, Egglesston JC and Coffey DS (1984) A new method to assess metastatic potential of human prostate cancer: relative nuclear roundness factor. *J Urol* **128**: 729–734

Finn CB, Luesley DM, Buxton EJ, Blackledge GR, Kelly K, Dunn JA and Wilson S (1992) Is stage I epithelial ovarian cancer overtreated both surgically and systemically? Results of a five-year cancer registry review. *Br J Obstet Gynecol* **99**: 54–58

Fuhrman SA, Lasky LC and Limas C (1982) Prognostic significance of morphologic parameters in renal cell carcinoma. *Am J Surg Pathol* **6**: 655–663

Fukuzawa S, Hashimura T, Sasaki, Yamabe H and Yoshida O (1995) Nuclear morphometry for improved prediction of the prognosis of human bladder carcinoma. *Cancer* **76**: 1790–1796

Gahlinger PM and Abramson JH (1995) *Computer programs for epidemiologic analysis: PEPI*. USD Inc: Stone Mountain, GA

Goff BA, Cuesta RS, Muntz HG, Fleischhacker D, Ek M, Rice LW, Nikrui N, Tamimi HK, Cain JM, Green BE and Fuller AF (1996) Clear cell carcinoma of the ovary: a distinct histologic type with poor prognosis and resistance to platinum-based chemotherapy in stage III disease. *Gynecol Oncol* **60**: 412–417

Haapasalo H, Collan Y, Seppa A, Gidlund A-L, Atkin NB and Pesonen E (1990) Prognostic value of ovarian carcinoma grading methods – a method comparison study. *Histopathology* **16**: 1–7

Haapasalo H, Atkin NB, Collan Y, Pesonen E and Paljrvi L (1991) Tumour ploidy, morphometry, histological and clinical features in ovarian carcinoma: mutual relations. *Anal Cell Pathol* **3**: 261–271

Jenison EL, Montag AG, Griffiths CT, Welch WR, Lavin PT, Greer J and Knapp RC (1989) Clear cell adenocarcinoma of the ovary: a clinical analysis and comparison with serious carcinoma. *Gynecol Oncol* **32**: 65–71

Kennedy AW, Biscotti CV, Hart WR and Webster KD (1989) Ovarian clear cell adenocarcinoma. *Gynecol Oncol* **32**: 342–349

Kennedy AW, Biscotti CV, Hart WR and Tauson LJ (1993) Histologic correlates of progression-free interval and survival in ovarian clear cell adenocarcinoma. *Gynecol Oncol* **50**: 334–338

Klotz JK and Cheung YK (in press) A linked list for the Wilcoxon signed rank test. *J Nonparametric Stat*

Miller BE, Lavia LA and Horbelt DV (1991) The prognostic value of image analysis in ovarian cancer. *Cancer* **67**: 1318–1321

Montag AG, Jenison EL, Griffiths CT, Welch WR, Lavin PT and Knapp RC (1989) Ovarian clear cell carcinoma: a clinicopathologic analysis of 44 cases. *Int J Gynecol Pathol* **8**: 85–96

Nativ O, Sabo E, Raviv G, Medalia O, Moskovitz B and Goldwasser B (1995) The role of nuclear morphometry for predicting disease outcome in patients with localized renal cell carcinoma. *Cancer* **76**: 1440–1444

O'Brien MER, Schofield JB, Tan S, Fryatt I, Fisher C and Wiltshaw E (1993) Clear cell epithelial ovarian cancer (mesonephroid): bad prognosis only in early stages. *Gynecol Oncol* **49**: 250–254

Partin AW, Walsh AC, Pitocock RV, Mohler JL, Epstein JI and Coffey DS (1989) A comparison of nuclear morphometry and Gleason grades: a predictor of prognosis in stage A2 prostate cancer: a critical analysis. *J Urol* **142**: 1254–1258

Petru E, Lahousen M, Tamussion K, Pickel H, Stranzl H, Stettner H and Winter R (1994) Lymphadenectomy in stage I ovarian cancer. *Am J Obstet Gynecol* **170**: 656–662

Pound CR, Partin AW, Epstein JI, Simons JW and Marshall FF (1993) Nuclear morphometry accurately predicts recurrence in clinically localized renal cell carcinoma. *Urology* **42**: 243–248

Rodenburg CJ, Cornelisse CJ, Hermans J and Fleuren GJ (1988) DNA flow cytometry and morphometry as prognostic indications in advanced ovarian cancer: a step forward in predicting the clinical outcome. *Gynecol Oncol* **29**: 176–185

Serov SF, Scully RE and Jobin LH (1973) Histologic typing of ovarian tumours in international histological classification of tumours. *World Health Organization, Geneva, No. 9*, pp. 37–42

Sharkey FE, Pavlak RJ and Greiner AS (1983) Morphometric analysis of differentiation in human breast carcinoma. *Arch Pathol Lab Med* **107**: 406–410

Wagner TMU, Adler A, Sevelda P, Asssmann I, Knepflf CF, Czerwenka K and Heinzl H (1994) Prognostic significance of cell DNA content in early-stage ovarian cancer (FIGO stage I and II/A) by means of automatic image cytometry. *Int J Cancer* **56**: 167–172

Williams DA (1988) Test for differences between several small proportions. *Appl Stat* **37**: 421–434

*© Cancer Research Campaign 1999*
